# Evaluating the Effectiveness of Cognitive Interventions for Healthy and Mild Cognitive Impairment Adults: A Comprehensive Umbrella Meta-Analysis

**DOI:** 10.1155/jare/4397025

**Published:** 2025-06-16

**Authors:** Giuseppe Forte, Francesca Favieri, Ilaria Corbo, Ilaria Chirico, Rabih Chattat, Anna Maria Della Vedova, Anna Pecchinenda, Maria Casagrande

**Affiliations:** ^1^Department of Dynamic and Clinical Psychology, and Health Studies, Sapienza University of Rome, Rome, Italy; ^2^Department of Psychology, University of Bologna, Bologna, Italy; ^3^Department of Clinical and Experimental Sciences, University of Brescia, Brescia, Italy; ^4^Department of Psychology, Sapienza University of Rome, Rome, Italy

**Keywords:** cognitive interventions, effectiveness, healthy aging, mild cognitive impairment, umbrella meta-analysis

## Abstract

Extensive research indicates that cognitive interventions can lead to a general improvement in cognitive functioning throughout the lifespan. In this study, we evaluate the causal evidence supporting this relationship in healthy older adults and older adults with mild cognitive impairment (MCI) by means of an umbrella meta-analysis of meta-analyses. The meta-analytic studies were identified through systematic searches in electronic databases (CINAHL, Cochrane Library, PsycINFO, PubMed, Scopus, and Web of Science) and were included in this umbrella meta-analysis if they examined the effects of cognitive interventions, not mixed with other approaches, in healthy older adults and individuals with MCI. Of the 9734 publications that were screened, 25 met the inclusion criteria and reported comprehensive data suitable for statistical analysis. Findings showed that although the effect-sizes across studies were variable, they were consistently positive, indicating a significant impact of different cognitive interventions on global cognitive functioning, memory, executive functions, visuospatial ability, and processing speed compared to control groups. This finding suggests that the efficacy of cognitive treatments is the best option for preclinical forms of aging, such as MCI. The underlying mechanisms of the observed improvements and their implications for further studies and clinical practice are discussed.

## 1. Introduction

The World Health Organization has reported that the processes of brain aging and aging-related neurodegenerative disorders are posing a significant challenge for the sustainability of health systems [1]. The latest international reports highlight the aging trend of the worldwide population and the prevalence of individuals aged 65 and above, which is projected to reach one in six by 2050 [2]. In this context, there is a notable emphasis on positive aging, which encompasses various dimensions of health, such as physical, functional, social, and psychological well-being [3, 4]. Along the continuum from physiological to pathological aging, one of the primary areas of investigation is the decline of cognitive functioning and the processes of brain aging. Accordingly, the current perspectives highlight the necessity of early interventions to prevent or delay cognitive decline and to mitigate its adverse consequences, such as loss of autonomy and poorer quality of life (QOL) [5]. Because the aging brain can benefit from an exploitable neural reserve, which is modulated by numerous factors such as genetic and epigenetic dynamics, education and experience-driven adaptation, lifestyle, and intelligence, cognitive enhancement appears to be a functional way to increase QOL [6–11]. In this regard, the research is focused on pharmacological and nonpharmacological approaches to contrast the changes in cognitive functions occurring with typical and atypical aging [12]. However, the lack of effective pharmacological interventions for the treatment and prevention of age-related cognitive impairment and of the different forms of dementia, a focus on nonpharmacological interventions appears to be a priority [13–15].

Nonpharmacological cognitive approaches, such as cognitive training, real-world interventions, and psychosocial approaches, offer reliable alternatives [15–17] and are strongly recommended for maintaining cognitive functions in healthy aging and in reducing cognitive decline in mild cognitive impairment (MCI [18]). Indeed, the beneficial impact of cognitive interventions is now widely acknowledged both for the cognitive decline of the typical aging and for the cognitive impairment in dementia [19]. The effectiveness of cognitive interventions is also documented by neuroscientific research and neuroimaging studies, indicating an increased activation in multiple brain regions, including the hippocampus, the right inferior parietal lobe, the frontoparietal network, and the occipitotemporal areas [20]. This evidence suggests that cognitive training of specific functions, whether employed as a standalone intervention or in conjunction with pharmacological treatments, may enhance cognitive benefits.

Importantly, the main conclusions of many narrative [21–23] and systematic reviews [24–26], as well as in meta-analyses [27–29], are as follows: (1) cognitive interventions have been demonstrated to enhance cognitive performance in both healthy older adults and adults with MCI [29–32], (2) the impact of such interventions appear to be beneficial across multiple cognitive domains [28, 29, 33], and (3) most studies report a large degree of heterogeneity in intervention characteristics, which should be further explored in order to define the terms of efficacy.

This large heterogeneity is due to multiple factors such as that extensive meta-analyses (e.g., [34–37]) entail summarizing the effect size (ES) of studies focused on cognitive interventions characterized by different protocols [20, 34, 38] and diverse population-range, from healthy older adults individuals to those with mild, moderate, or severe cognitive impairment [39–41]. Therefore, despite the large number of primary studies available, the relative benefits of the different cognitive interventions for the aging population are still unclear, especially when considering that a cognitive intervention may be more effective to improve the general cognitive functioning in healthy aging, whereas another may be more effective in cases of MCI. One way to overcome these relative limitations is provided by an umbrella meta-analysis, which is regarded by Booth et al. [42] as the optimal methodology for synthesizing the extant knowledge on a given topic. Consequently, there has been a proliferation of umbrella reviews and, most recently, meta-analysis of meta-analyses [43–45]. Umbrella meta-analyses represent relatively new approach, which summarizes and stratifies the quality and strength of evidence from individual meta-analysis, and it relies on recalculating the meta-analytic estimates while adopting uniform criteria among studies, which allows for consistent evidence stratification [46]. Moreover, according to Ciria et al. [47], umbrella meta-analysis is an effective tool for informing public health policies and recommendations, with guidelines that are firmly grounded in scientific evidence.

Accordingly, we set out to conduct an umbrella meta-analysis aimed at addressing the state of the art on the effectiveness of cognitive interventions in healthy aging and in MCI by exploring and examining the meta-analytic published work. The objective is to determine whether the claims regarding the benefits of cognitive interventions in aging are supported by solid and reliable empirical evidence, when assessed adopting homogenous criteria. This approach is considered to provide reliable proofs and represents the current gold standard for ascertaining causal links between interventions and outcomes. By adopting a common framework, we aimed to provide clinicians and researchers with valuable insights into the most effective interventions for specific conditions (i.e., healthy aging, MCI) and cognitive functions. Furthermore, by evaluating the effectiveness of cognitive interventions, their strengths and limitations, the outcomes of the present umbrella meta-analysis contribute to scientific progress and can guide policies and guidelines in the field.

## 2. Materials and Methods

### 2.1. Protocol and Registration

This umbrella meta-analysis was registered on PROSPERO (CRD42023447523; registered data: 02/08/2023) according to Preferred Reporting Items for Systematic Reviews and Meta-Analyses (PRISMA) guidelines [48].

### 2.2. Research Strategies

**Figure 1:**
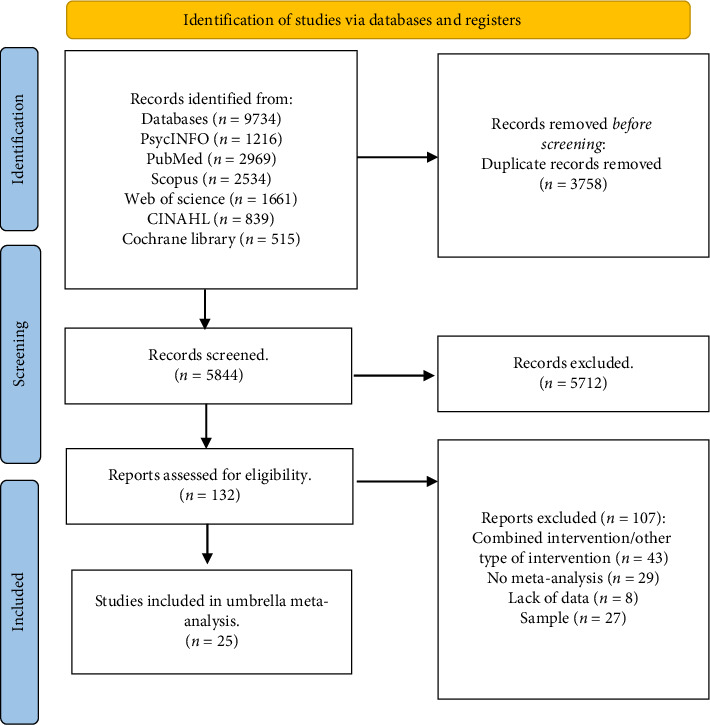
Flowchart, PRISMA statement 2020.

This umbrella meta-analysis was conducted by selecting articles in peer-reviewed journals using the databases PsycINFO, Web of Science, CINAHL, Scopus, PubMed, and Cochrane Library. The first systematic search was conducted in May 2023. The last search for checking update was performed on August 1st, 2024. The script used consistently across all databases was as follows: *(“mild cognitive impairment” OR healthy and (elderly or old^∗^)) and (Cognitive stimulation therapy or Cognitive Stimulation or CST or reality orientation or memory therapy or memory groups or memory support or memory stimulation or global stimulation or cognitive psychostimulation OR psychosocial intervention OR cognitive intervention OR cognitive remediation OR cognitive training OR cognitive rehabilitation OR cognitive stimulation OR neuropsychological rehabilitation OR reminiscence) and (review or meta-analysis or metanalysis).* Restrictions were to academic publications in English and Italian languages. Meta-analytic studies of empirical evidence on cognitive interventions in the human population were included without restrictions regarding gender and ethnicity. The search strategies are reported in Figure 1.

### 2.3. Eligibility Criteria

After the screening procedure, duplicates were eliminated using the Zotero software. The list of potential articles produced by the systematic research was revised. Then, nonrelevant studies were excluded based on the title and abstract. Two authors (Ilaria Corbo and Francesca Favieri) independently assessed the records, with any discrepancies resolved by a third reviewer (Giuseppe Forte). Following this, a further selection was conducted by evaluating the full-text adhering to predefined inclusion/exclusion criteria. All authors (Ilaria Corbo, Francesca Favieri, Giuseppe Forte, Ilaria Chirico, Anna Maria Della Vedova, Anna Pecchinenda, and Maria Casagrande) independently screened full texts using a standardized extraction form, and any discrepancies were resolved through team discussion.

The following inclusion criteria were adopted: (i) meta-analysis reporting ESs of cognitive interventions in older adults (i.e., cognitive stimulation therapy, computerized cognitive intervention, reality orientation, cognitive intervention, cognitive remediation, cognitive training, neuropsychological rehabilitation, or reminiscence therapy); (ii) studies involving either healthy older adult population or individuals diagnosed with MCI, due to the adoption of such kind of intervention in multiple cognitive condition; (iii) studies focused on multiple cognitive domains targeted by the intervention and assessed by reliable neuropsychological tools (e.g., global functioning, executive functions, memory, processing speed, and attention), with the aim to cover the multiple cognitive outcomes that usually are influenced by cognitive intervention. Exclusion criteria provided are as follows: (i) studies on people with dementia diagnosis, from mild to severe or co-occurrence of other clinical conditions and psychiatric disorders (e.g., traumatic brain injury, metabolic disorders, cardiovascular disorders, chronic disorders, and cancer), because the effect of the established clinical condition or profound cognitive impairment would increase the heterogeneity of the ES and related evidence; (ii) studies integrating cognitive interventions in a multiple approach (e.g., psychosocial approach and physical activity programs) because hybrid approaches would not allow to verify the specific effectiveness of the cognitive approach; (iii) studies assessing cognitive performance with self-reported tools or evaluation by caregivers, as poorly reliable and nonobjective outcome data. Also, reviews, clinical trials, cross-sectional and longitudinal studies, editorials, comments, and replies were likewise excluded.

### 2.4. Data Collection

According to PICOS approach [49, 50], the following information was extracted from each included meta-analysis: author and year of publication, type of intervention, duration of single intervention, duration of intervention, number of session, population, age, outcome, number of qualitative studies, main findings, type of publication bias, outcome of unique meta-analysis, general cognitive domain, number of quantitative studies, sample size, type of ES, ES mean, lower and higher confidence interval (CI) of ES, *p* value, heterogeneity (*I*^2^), and direction of findings. All extracted data are summarized in Supporting Table 1b.

### 2.5. Quality Assessment

The ROBIS checklist [51], specifically developed for assessing the risk of bias in systematic reviews rather than primary studies, was used in this work. The tool consists of four specific domains: (i) study eligibility criteria, (ii) identification and selection of study, (iii) data collection and study appraisal, and (iv) syntheses and findings. Each domain consists of a series of questions with a five-point response scale (*yes*, *probably yes*, *probably no*, *no*, and *not reported*). The sum of the scores for each question within a domain provides the risk of domain-specific bias, which can be classified as low, unclear, or high. At the end of the assessment of the four specific domains, there is a general domain consisting of three questions that assess the risk of general bias (See Supporting Table 1a).

### 2.6. Statistical Analysis

Standardized mean differences (SMD) between treatment and control groups at the meta-meta-analytic level with 95% CI were calculated to reduce the heterogeneity in the measurement instruments employed in the primary studies. According to Cohen [52], an SMD of 0.2 is considered a small ES, 0.5 a moderate ES, and 0.8 a large ES. We conducted separate meta-meta-analyses for each cognitive domain (global cognitive functions, memory, executive functions, attention, visuospatial ability, processing speed, and language) as well as a general meta-analysis including all domains. ES was reported according to the original publication, while SE was calculated according to the formula: (upper limit – lower limit)/3.92 (Cochrane Handbook for Systematic Reviews of Interventions). The amount of heterogeneity between studies was quantified using the *I*^2^ statistic. Outlier analyses were conducted using the leave-one-out approach [53], and the influence on the remaining heterogeneity of the overall effects was assessed. After identifying and removing outliers, the ES was recalculated. Finally, the association between mean ESs and other moderator variables was continuously examined by conducting moderator analyses and/or meta-regression analyses with random effects models. All analyses were carried out using R Version 4.0.0 with the packages ‘meta,' ‘metafor,' and ‘dmetar' [54–56].

### 2.7. Primary Study Overlap

A critical issue in the analysis of meta-meta-analytic data is the possibility of overlapping primary studies in individual meta-analyses. To ensure statistical independence of ESs, each primary study must be represented only once in the final analyses. There are several strategies to deal with overlapping primary studies [57–59]. However, since most strategies were not applicable to the present analysis (See [60]), we followed the indications of Tuerk et al. [61] to visualize the degree of overlap, created a citation matrix for each cognitive domain separately, and calculated the corrected covered area (CCA [62]). CCA was computed using the following formula: CCA = (*N* − *r*)/(*r* ∗ *c*) − *r*, where *r* represents the number of primary studies, *c* is the number of meta-analyses, and *N* is the number of times the studies appeared in the reviews. Therefore, the CCA represents a measure of the overlap (relative coverage) of primary studies in the included meta-analyses. Meta-analyses with high overlap were first included in a general ES estimation and then excluded for additional analyses to examine the amount of influence on the overall effects.

## 3. Results

### 3.1. Results of the Search

The screening process yielded a total of 9734 articles. After the first step, 3758 duplicates were eliminated. Title and abstract screening excluded 5844 nonrelevant studies and 132 full texts were evaluated. After the screening process, 25 meta-analyses ultimately met our inclusion criteria and were included in the qualitative synthesis (see Figure 1).

### 3.2. Qualitative Analyses

#### 3.2.1. Participant Data

Twenty-five meta-analyses were included, and 111 ESs were compared. The sample consisted of 80,910 healthy and older adult participants with MCI, ranging from 17 [63] to 8783 [64]. Participants included in the meta-analyses were individuals older than 50 years (See Supporting Table 1b).

#### 3.2.2. Outcome Data

Most meta-analyses (24 out of 25) examined the effectiveness of cognitive treatments on the primary outcome for cognition, while one meta-analysis examined the effectiveness of cognitive treatments in relation to QOL [65].

Twenty-six meta-analyses analyzed the efficacy of cognitive treatments for global cognitive functioning [20, 28–30, 32, 33, 37, 38, 64–74]. Forty-three meta-analyses evaluated cognitive treatment efficacy related to performance in memory domain [15, 17, 28, 29, 32–34, 36–38, 65, 67, 70–72].

Twenty-three meta-analyses examined the effectiveness of cognitive treatments in relation to performance in executive functions' domain [17, 28, 29, 32–34, 36–38, 65, 67, 70–72]. Five meta-analyses evaluated cognitive treatment efficacy for attention [28, 32, 33, 37, 72]. Six meta-analyses analyzed cognitive treatment efficacy in relation to performance in the visuospatial ability domain [28, 29, 32, 33, 37]. Four meta-analyses analyzed cognitive treatment efficacy for performance in processing speed [17, 33, 36, 37]. Finally, four meta-analyses investigated cognitive treatment efficacy in relation to performance in the language domain [29, 32, 38].

#### 3.2.3. Intervention Data

Eight meta-analyses assessed cognition-based interventions [20, 28, 65–67, 69, 75, 76]. This type of intervention includes cognitive stimulation, cognitive rehabilitation, cognitive training, and memory training. Eight meta-analyses assessed computerized cognition-based interventions [28–30, 33, 37, 71, 74]. Seven studies used virtual-reality interventions [29, 32, 38, 70, 72–74]. Two meta-analyses assessed only cognitive training [63, 64]. Six meta-analyses were on memory/mnemonic training [15, 20, 28, 34, 67, 75]. One study used a real-world intervention [17]. An overview of the main characteristics of included meta-analyses is presented in Table 1 and Supporting Table 1b.

### 3.3. Methodological Quality of Included Meta-Analysis

#### 3.3.1. Results of Risk of Bias

**Figure 2:**
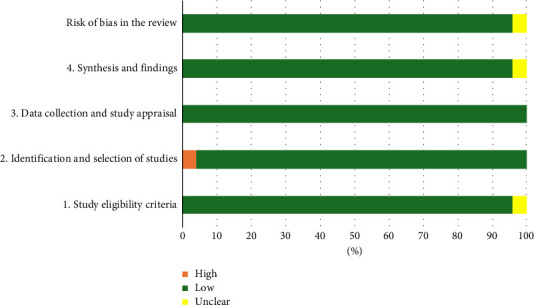
Results of ROBIS checklist.

The risk of bias was independently assessed by three authors (IC, FF, and Giuseppe Forte). The results revealed a low risk of bias in most of the included studies. Only two studies reported critical issues. Campos and Vasconcellos [34] showed an unclear risk of bias in Domain 1, a high risk of bias in Domain 2, and a general unclear risk of bias. For Martin et al. [63], an unclear risk of bias was found in Domain 4. It is interesting to note that, generally, eligible meta-analyses on this topic are characterized by high standards for data collection (Domain 3), with a good control of the data (in terms of outcomes and statistical analyses) to select and include in the synthesis. Few limits emerged in study eligibility (Domain 1) probably due to, although with minimum risk, the presence of weak or unclear selection criteria for the included works (See Figure 2 for details).

#### 3.3.2. Primary Study Overlap

The CCA represents the primary study overlap and ranges from moderate (i.e., memory, cognitive function, and executive functions) to high (i.e., language). In these 25 meta-analyses, 981 total studies were included, of which 556 were unique (see Table 2).

According to Pieper et al. [62], a CCA between 0 and 5 indicates slight overlap, a CCA of 6–10 indicates moderate overlap, a score between 11 and 15 indicates high overlap, and a score greater than 15 indicates very high overlap.

### 3.4. Quantitative Analyses

#### 3.4.1. Included Data

Seventy-five ESs were compared in the quantitative analysis, whereas 18 of the meta-analyses included in the qualitative analysis underwent a mediation process, in which the ESs of the meta-analyses with the same outcome were mediated with each other. More specifically, for Bonnèchere et al. [37], Vaportzis et al. [17], Li et al. [65], Lampit et al. [33], Zhang et al. [71], Zhong et al. [72], and Gomez-Caceres et al. [32], the ESs of the meta-analyses for executive functioning and working memory were mediated. For Li et al. [75] and Zhong et al. [72], the ESs of the meta-analyses for global cognitive functioning were mediated. For Li et al. [65], Lampit et al. [33], Campos and Vasconcellos [34], Zhong et al. [72], Gomez-Caceres et al. [32], and Martin et al. [63], the ESs of the meta-analyses for memory were mediated. In addition, for Martin et al. [63], the included meta-analyses were mediated according to the population of interest (healthy and MCI).

#### 3.4.2. Effect of Interventions

Generally, our meta-analysis showed a combined effects size, considering all cognitive domains, of SMD = 0.29 (95% CI, 0.21 to 0.36; *z* = 7.17, *p* < 0.001) with *τ*2 = 0 and *I*^2^ = 0%, indicating no heterogeneity between results from individual meta-analyses (Figure 2). Supporting Table 1b in Supporting Information provides a summary of each outcome's results.

##### 3.4.2.1. Global Cognitive Functions

**Figure 3:**
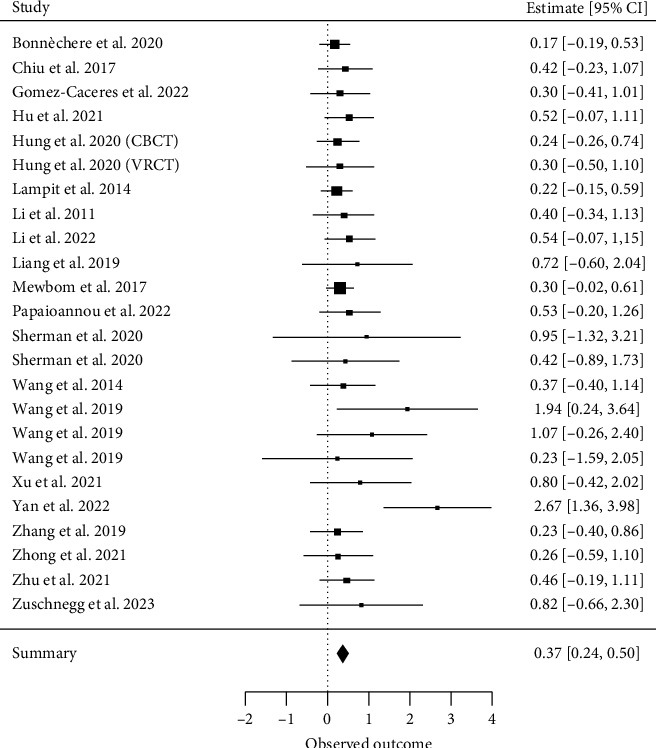
Global cognition.

Twenty-four meta-analyses examining global cognitive functions [20, 28–30, 32, 33, 37, 38, 64–75] met our inclusion criteria for a quantitative analysis. A total of 222 primary studies were included, with a CCA of 6%, indicating no significant overlap. The overall effect resulted in an ES of SMD = 0.37 (95% CI, 0.24 to 0.51; *z* = 5.45, *p* < 0.001) with *τ*2 = 0 and *I*^2^ = 0%, indicating no heterogeneity between the results of individual meta-analyses (Figure 3).

##### 3.4.2.2. Memory

Seventeen meta-analyses investigating memory [15, 28, 29, 32–34, 36–38, 63, 65, 67, 70–72] met our inclusion criteria for a quantitative analysis. A total of 116 primary studies were included, with a CCA of 8%, indicating no significant overlap.

**Figure 4:**
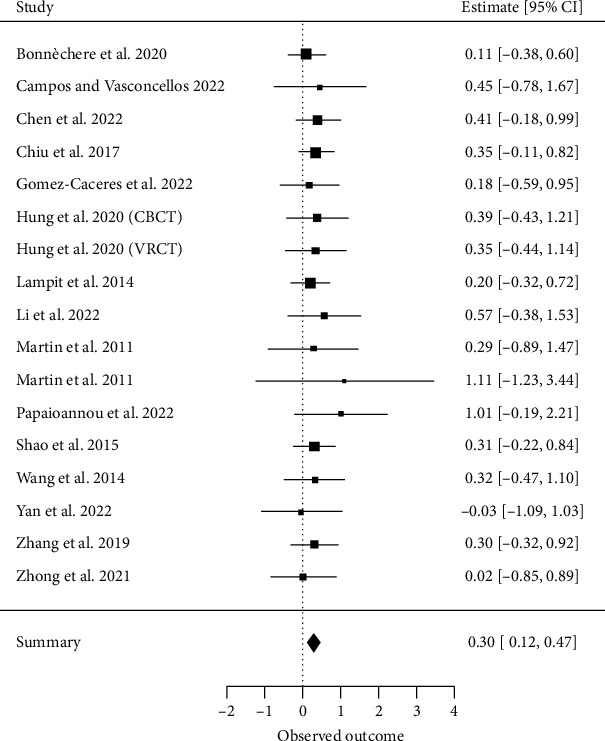
Memory.

The overall effect resulted in an ES of SMD = 0.30 (95% CI, 0.12 to 0.47; *z* = 3.35, *p* < 0.001) with *τ*2 = 0 and *I*^2^ = 0%, indicating no heterogeneity between the results of individual meta-analyses (Figure 4).

##### 3.4.2.3. Executive Functions

**Figure 5:**
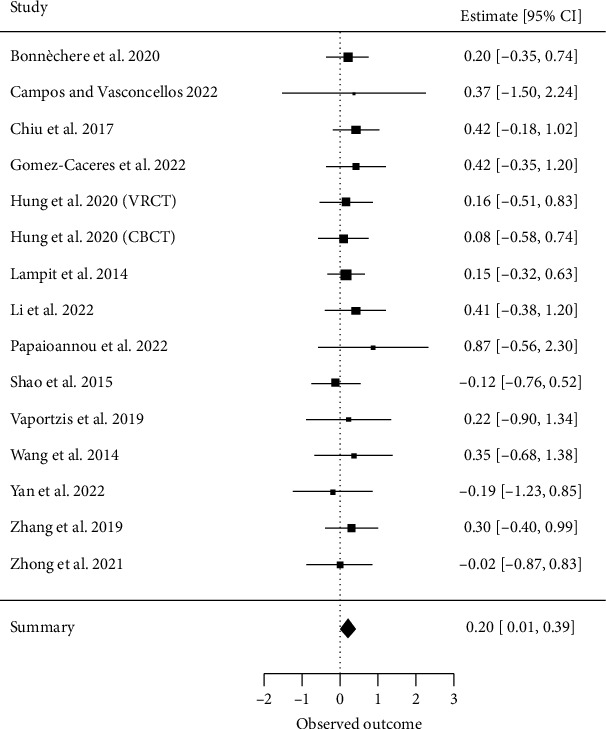
Executive functions.

Fifteen meta-analyses investigating executive functions [17, 28, 29, 32–34, 36–38, 65, 67, 70–72] met our inclusion criteria for quantitative analysis. A total of 103 primary studies were included, with a CCA of 9%, indicating no significant overlap. The overall effect resulted in an ES of SMD = 0.20 (95% CI, 0.01 to 0.39; *z* = 2.07, *p*=0.04) with *τ*2 = 0 and *I*^2^ = 0%, indicating no heterogeneity between the results of individual meta-analyses (Figure 5).

##### 3.4.2.4. Attention

**Figure 6:**
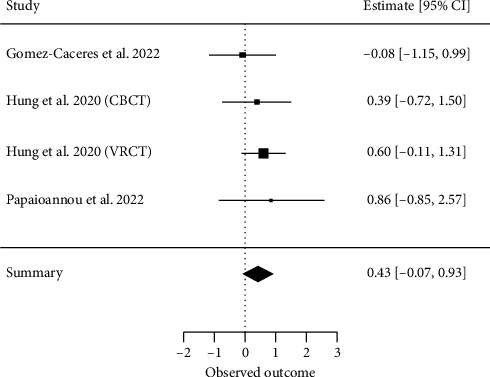
Attention.

Five meta-analyses investigating attention [28, 32, 33, 37, 72] met our inclusion criteria for a quantitative analysis. The overall effect resulted in an ES of SMD = 0.06 (95% CI, −0.22 to 0.36; *z* < 1, *p*=0.64) with *τ*2 = 0.02 and *I*^2^ = 15.08%, indicating low heterogeneity between the results of individual meta-analyses (Figure 6). CCA was 22%, indicating a moderate overlap of the primary study.

##### 3.4.2.5. Visuospatial Ability

**Figure 7:**
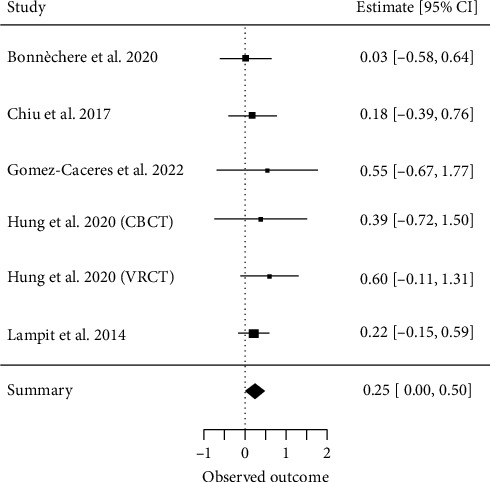
Visuospatial ability.

Six meta-analyses investigating visuospatial ability [28, 29, 32, 33, 37] met our inclusion criteria for a quantitative analysis. A total of 25 primary studies were included, with a CCA of 18%, indicating low overlap. The overall effect resulted in an ES of SMD = 0.25 (95% CI, 0.004 to 0.50; *z* = 1.99, *p*=0.04) with *τ*2 = 0 and *I*^2^ = 0%, indicating no heterogeneity between the results of individual meta-analyses (Figure 7).

##### 3.4.2.6. Processing Speed

**Figure 8:**
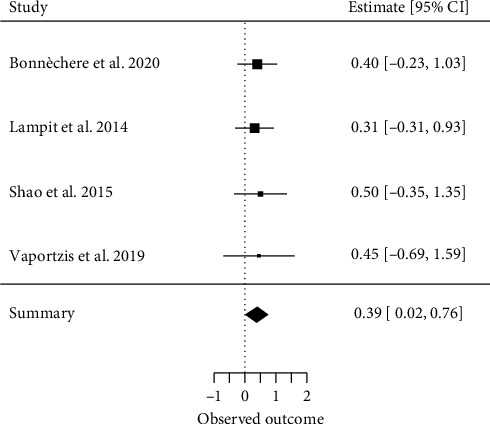
Processing speed.

Four meta-analyses investigating processing speed [17, 33, 36, 37] met our inclusion criteria for a quantitative analysis. A total of 40 primary studies were included, with a CCA of 38%, indicating moderate overlap. The overall effect resulted in an ES of SMD = 0.39 (95% CI, 0.02 to 0.76; *z* = 2.08, *p*=0.04) with *τ*2 = 0 and *I*^2^ = 0%, indicating no heterogeneity between the results of individual meta-analyses (Figure 8).

##### 3.4.2.7. Language

**Figure 9:**
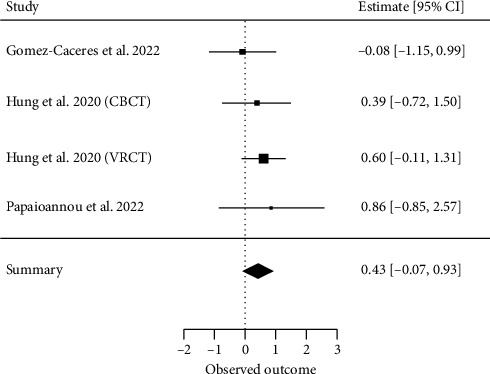
Language.

Four meta-analyses investigating language [29, 32, 38] met our inclusion criteria for a quantitative analysis. A total of nine primary studies were included, with a CCA of 61%, indicating higher overlap. The overall effect resulted in an ES of SMD = 0.43 (95% CI, −0.06 to 0.93; *z* = 1.69, *p*=0.08) with *τ*2 = 0 and *I*^2^ = 0%, indicating no heterogeneity between the results of individual meta-analyses (Figure 9).

##### 3.4.2.8. Subgroup Analyses Considering Cognitive Status

Meta-analyses reporting ESs of MCI subgroups resulted in SMD of 0.36 (95% CI, 0.21 to 0.51; *z* = 4.79; *p*=0.0001) with *τ*2 = 0 and *I*^2^ = 0%, indicating no heterogeneity, and meta-analyses of healthy participants yielded an SMD of 0.23 (95% CI, 0.12 to 0.34; *z* = 4.11; *p*=0.001) with *τ*2 = 0 and *I*^2^ = 0%, indicating no heterogeneity. Lastly, meta-analyses of mixed MCI and healthy participants yielded an SMD of 0.33 (95% CI, 0.16 to 0.51; *z* = 3.79; *p*=0.0002) with *τ*2 = 0 and *I*^2^ = 0%, indicating no heterogeneity. In subgroup analyses, no significant differences were found between MCI, healthy participants, or mixed groups.

## 4. Discussion

In this umbrella meta-analysis, we examined the current state of the art on whether cognitive interventions lead to cognitive gains in both healthy older adults and in individuals with MCI. We analyzed 25 meta-analyses, encompassing a total of 981 primary studies and 80,910 participants (54,664 healthy adults and 15,021 adults with MCI). Our findings show the potential cognitive benefits of cognitive interventions for both healthy and MCI individuals, despite the medium ESs yield higher values for interventions with MCI than with healthy populations (SMD = 0.36 vs. SMD = 0.23). Subgroup analyses revealed no significant differences in the beneficial effect between the groups, indicating that this type of intervention would be well-received and may be effective in a range of conditions, from physiological decline to MCI. Moreover, these findings are in keep with the conclusions of previous narrative works (e.g., Tsai and Shen [77]) that include cognitive activities in the list of factors that can delay and counteract the progression to severe cognitive decline and dementia. These factors include physical activities, diet, and positive relationships. This is of particular importance given the emphasis placed by researchers and clinicians on the necessity to identify strategies to prevent pathological aging and to strengthen cognitive functions (e.g., which helps to mitigate brain damage and to reduce the risk for health and autonomy).

The frame in which this evidence is presented is the current lack of FDA-approved agents for the treatment of MCI, as well as the necessity to prevent the deterioration of cognitive functions associated with brain aging. The inherent uncertainty about the progression of MCI and the lack of effective treatment agents increases the potential impact of the present findings, especially as experts have pointed out the many limitations in the available knowledge on cognitive decline and MCI [78], and previous guidelines and recommendations are no longer considered valid. These limitations are often highlighted in national guidelines in which they analyze all types of pharmacological and nonpharmacological interventions in order to get a better overview of effective treatments to cope with dementia, for example, reminiscence therapy, pet therapy, transcranial stimulation, and individual and group cognitive training [79]. In this sense, systematic work such as umbrella reviews and umbrella meta-analyses can help by providing stronger evidence and/or by clarifying the boundaries of the available evidence used to structure intervention protocols and assist in the implementation of policies and recommendations.

Our findings indicate that cognitive interventions enhance general cognitive performance (ES = 0.37), with notable impacts on memory, executive functions, processing speed, language, and visuospatial ability. The ESs ranged from modest to high (0.07–0.43), with low heterogeneity. Importantly, the strength of these conclusions is that they are based on a comprehensive overview of the scientific evidence accumulated over the past 2 decades. There is compelling evidence of a growing interest on how to best design interventions for specific cognitive domains, such as memory and executive functions, as documented by the higher number of quantitative syntheses on these domains and the average number of primary studies included. These domains have historically been linked to cognitive decline and appear to be the most affected in pathological cognitive decline, such as Alzheimer's disease or frontotemporal dementia [80–84]. Therefore, scholars are interested not only in the prodromal characteristics of a pathological decline but also in how to maintain and improve the functionality of the affected cognitive domains. Moreover, there is a growing concern that memory loss is becoming more severe, particularly in light of the long-standing view that memory is inextricably linked to the self, and how it interacts with the world in daily life, which is seen as a key indicator of autonomy and QOL [85, 86]. However, we share the view that loss of memory does not equate to a global loss of self [87], except when autobiographical memory is involved [88]. In addition, we would like to emphasize that other cognitive functions are equally important and could be adequately investigated as valid targets of cognitive interventions to prevent or slow down pathological cognitive decline [36, 37].

One question that frequently arises and motivates research is why these interventions are effective. Neuroimaging studies provide some useful insights to a certain extent. Cognitive interventions appear to be effective due to their repetitive nature and progressive difficulty, which can lead to neuroplastic changes. These changes include an increment in synaptic density, enhanced connectivity between neural networks, and the formation of new neural pathways [89–91]. For example, older adults who undergo cognitive treatment show increased gray matter volume in the temporal regions and hippocampus, as well as increased thickness in the right insula and orbitofrontal and fusiform cortices [92, 93]. Importantly, the goal of cognitive interventions is not to impart new skills but rather to improve cognitive functions that may be impaired [28]. Furthermore, cognitive training may facilitate the development of metacognitive abilities, enabling individuals to employ more effective strategies for problem-solving and information processing, which are essential for maintaining individual autonomy. Neuroimaging evidence (for a review see Beishon et al.'s study [94]) also shows that cognitive training in older adults with dementia and MCI has a positive impact on the default mode network (DMN). The DMN plays a pivotal role during resting state activity, connecting areas affected by Alzheimer's disease (e.g., precuneus, medial temporal lobe, and parietal and prefrontal cortex). Alterations in the DMN connectivity are associated with pathological aging and cognitive decline, whereas maintaining optimal DMN connectivity serves as a protective factor against disease progression. Therefore, cognitive training can reorganize connectivity in these areas and the DMN in both older adults with Alzheimer's disease and in adults with MCI (Beishon et al. [94]). There is also evidence that memory training in older adults with Alzheimer's disease increases functional connectivity in the middle temporal gyrus, precuneus, and occipital cortex (e.g., Hampstead et al.'s study [95]). Conversely, individuals with greater cortical thickness in the hippocampus at baseline, a region most affected by Alzheimer's disease, benefit more from cognitive treatment [96]. Other studies in healthy older adults have shown that cognitive interventions can lead to increased hippocampal volumes and regional increases in cortical thickness [94]. Cognitive interventions are also effective in improving daily living skills [97, 98], which tend to decline because of physiological and, above all, pathological deterioration [99]. Furthermore, cognitive interventions impact cognitive reserve, which is a crucial element in cognitive rehabilitation. These interventions may be regarded as a form of “late-life education” helping individuals in coping with the effects of pathological aging [100]. Such interventions enhance neural and cognitive reserve, thereby reducing the risk of dementia [37, 101].

As a final point, we should acknowledge advantages and limitations of the present study. Among the advantages, we would like to point out that individual meta-analyses frequently fail to cite the original sources of evidence or pertinent primary data, which could prove invaluable in identifying the strengths and limitations of each intervention for each condition. Merge and test the ESs of the quantitative summary with similar characteristics and outcomes may point out more precise evidence on the intervention's efficacy. Single meta-analyses on similar topics are leading to a progressive accumulation of redundant evidence, characterized by low to moderate ES, and by evidence based on low statistical power, resulting in estimates with low precision and stability. Meta-analyses often report substantial ESs, but these results are derived from different studies, which hamper direct comparison between the meta-analyses, causing, in some cases, an overestimation of evidence [102]. Indeed, primary studies exhibit heterogeneity in their results; some report higher effectiveness of cognitive stimulation [28, 29, 33], while others do not consistently support this hypothesis [17, 72]. The umbrella meta-analysis help in covering these limitations. As confirmed by our study, outcomes are identified with disparate instruments and within the same domain, and samples are characterized by differences (e.g., in the criteria used to diagnose MCI [103]) that are difficult to control in the meta-analytic synthesis. In fact, the standardization of certain aspects upon inclusion in the meta-analyses enables a comparison between different studies. However, this process inevitably entails some loss of content, which must be considered as a potential limitation in subsequent analyses. Again, umbrella meta-analyses helped in identifying this aspect, allowing to highlight strength and limitation of a single meta-analysis. It is also important to consider that results of each quantitative synthesis are dependent on the quality of the included reports and the methodological decisions made. In this sense, the results from different meta-analyses cannot be taken as a comprehensive representation of the accumulated empirical evidence spanning years of research. Instead, they must be viewed as a selective compilation of that evidence. However, our findings interestingly indicate minimal heterogeneity, suggesting that a general effect—substantiated by empirical validation evidence—exists, allowing to read previous meta-analytic evidence under a clearer lens of interpretation. As outcome, this evidence further corroborates the notion that cognitive stimulation can positively influence cognitive functioning, thereby overcoming the limitations.

A significant limitation of this umbrella meta-analysis is the paucity of a clear theoretical model of the mechanisms involved, despite the abundance of published intervention studies, reviews, and meta-analyses. While previous research has provided valuable insight into potential neurobiological mechanisms, there is a need for a more comprehensive theoretical framework to explain the observed cognitive benefits [104, 105]. In fact, no hypothesis has been so far proposed to integrate the existing body of evidence on the cognitive benefits resulting from different intervention approaches, which make integration of different findings challenging. The multiple intervention procedures reported in Supporting Table 1b are provided in the supporting information, which are markedly different from one another, allowing the identification of a robust but unclear relationship, despite heterogeneity for aspects such as providers, strategies, duration, exercises, and procedures. Importantly, these considerations do not seem to influence the proven effectiveness of cognitive-oriented work in the older adults.

These aspects should be further analyzed, particularly considering technological advancements and the growing integration of immersive devices (e.g., virtual reality [70, 73]) and artificial intelligence. It is relevant to anticipate and address potential changes in intervention approaches to ensure the maintenance of optimal standards of benefit. We believe that individuals have the potential for self-improvement and the ability to live fully in later life. Cognitive interventions can significantly contribute to achieving this goal, both in a preventive and rehabilitative perspective, for both healthy and pathological populations. However, we must move beyond the notion of the brain as a muscle and contemplate the myriad facets of brain functions, as cognitive interventions alone may not be enough.

Although an umbrella meta-analysis can be considered the highest level of evidence in intervention research, it is also important to recognize the inherent limitations. First, although rigorous inclusion criteria are invaluable for mitigating the risk of heterogeneity and bias in the results, this work exclusively focused on the impact of cognitive interventions in healthy adults and adults with MCI. Accordingly, it excluded programs that were combined with other approaches (e.g., physical activity) [106]. Furthermore, it would be relevant to investigate the effectiveness of the interventions in the context of cognitive impairment, including more severe cognitive decline and dementia. Additionally, we should acknowledge possible limitations in the generalizability of our findings due to the lack of a comprehensive search for the gray literature. Moreover, although we provided a comprehensive description of the intervention patterns and outcomes of each meta-analysis (see Supporting Table 1b), we did not conduct a detailed statistical examination of the specific characteristics of each intervention and the diverse outcome indicators employed (e.g., different cognitive tasks). Consequently, definitive intervention recommendations regarding format, provider, duration, frequency, or setting cannot be provided. We also did not consider follow-up data, and thus the present evidence cannot speak to the long-term benefits of interventions on cognitive functions. This is one of the most frequently reported criticisms of the literature on intervention efficacy [107]. Additionally, the impact of cognitive stimulation on other health outcomes (e.g., depression, behavioral symptoms, and QOL) has not been evaluated. In this context, further evidence is necessary to determine how these individual factors interfere with the association between the cognitive condition and the efficacy of the intervention. Lastly, the findings should be interpreted with caution, as genetic and environmental factors may act as confounding variables, influencing brain reserve and cognitive reserve and affecting brain aging [108, 109].

## 5. Conclusion

In conclusion, when considering strengths and limitations, our findings provide evidence that cognitive interventions have a causal effect on cognitive enhancement. Until stronger evidence is available, it is recommended that these findings be incorporated into policies and guidelines that may prove beneficial to the population. As confirmed by this work, cognitive interventions offer not only cognitive benefits but also social and psychological advantages, which are yet to be fully explored. The findings of this umbrella meta-analysis can furnish insight on the design of targeted interventions for specific cognitive domains (e.g., memory, executive functions, and processing speed) suggesting the point on which implement programs in clinical and community settings as nonpharmacological strategies to prevent or delay cognitive decline.

All considering, it emerges the necessity to move away from the metaphor of the brain as a muscle to be trained and developed regardless of the type of “training”. Rather, it is promising to define comprehensive strategies considering multiple aspects of well-being and daily functioning from a biological, psychological, and social perspective, especially in a world characterizing by increasing aging and risk for dementia. Further research is needed on comprehensive theoretical models of the cognitive and neural mechanisms of these potential cognitive improvements induced by cognitive interventions. In this sense, our work represents a preliminary investigation, showing that cognitive interventions are effective. The next step is for further research to refine this knowledge by addressing one of the most important questions in science: “Why?.” These new findings could potentially be translated into social recommendations and evidence-based policies.

## Figures and Tables

**Table 1:** Main characteristics of included meta-analyses.

Author	Type of intervention	Characteristics of intervention	Characteristics of participants	No. of qualitative studies	Main finding	Outcome of unique meta-analysis
Bonnèchere et al. [37]	Commercial computerized cognitive games	No. of session: 16–60 Single intervention (min): 15–60 Total time: 5–60 h	HC Age: 62.3–81.9	16	Significant improvements were observed for processing speed, WM, EFs, and for verbal memory	- Visuospatial abilities - Attention - Processing speed - EFs - Verbal memory - WM - Overall
Campos and Vasconcellos [34]	MT	No. of session: 5–12 Single intervention (min): — Total time: —	HC Age: —	9	No effect of cognitive intervention (moderate and significant effect on incidental memory)	- Incidental memory - Semantic memory - WM - Episodic memory - Memory
Chen et al. [15]	MT	No. of session: 3–32 Single intervention (min): 60–180 Total time: 3.8–40 h	HC Age: 67.1–85.2	21	Immediate and long-term memory improvement	- Episodic memory (immediate effect)
Chiu et al. [28]	CBT	No. of session: 4–120 Single intervention (min): 15–120 Total time: 4–24 w	HC Age: 65.1–85.1	31	Cognitive training is effective	- Visual spatial ability - Overall cognitive functions - Memory - Attention - EFs
Gomez-Caceres et al. [32]	VRCT	No. of session: 8–100 Single intervention (min): 18–100 Total time: 4–24 w	MCI Age: 70.12–87.5	13	VR training is effective, but effects depend on domain and length of intervention	- Visuoconstruction - Language - WM - Immediate memory - Delayed memory - Global cognitive functioning - Attention - EFs
Hu et al. [30]	CCT	No. of session: 9–72 Single intervention (min): 40–160 Total time: 4–24 w	HC; SCD; MCI Age: 70.4–78.8	12	CCT good effectiveness especially in memory domain	- Overall cognitive functions
Hung et al. [29]	VRCT; CBCT	No. of session: — Single intervention (min): — Total time: —	HC; MCI Age: > 65	18	Both CBCT and VRCT effectiveness. VR training effectiveness on visuospatial abilities. CCT effectiveness on memory and language. Both the intervention have small effect size in EFs.	- Global cognitive function - Language - Visuospatial - EFs - Memory - Global cognitive function - Memory - EFs
Lampit et al. [33]	CBT	No. of session: 3–49 Single intervention (min): 15–120 Total time: 4–40 h	HC Age: 60.7–81.9	51	Improving of performance	- Visuospatial - Nonverbal memory - Attention - Working memory - Verbal memory - EFs - Processing speed - Global cognitive function
Li et al. [75]	CBT	No. of session: 3–103 Single intervention (min): 13–120 Total time: 2 ≥ 52 w	MCI Age: 61–78.9	20	MCI obtain moderate benefits in language, and receive mild benefits in episodic memory, semantic memory, EFs/WM, visuospatial ability, attention/processing speed, MMSE. The results also suggest that MCI benefit from the cognitive intervention in the follow-up data.	- MMSE - Overall cognition
Li et al. [65]	Computer-based intervention	No. of session: 4–24 Single intervention (min): 10–180 Total time: 4–24 w	MCI Age: 69.9–77.2	17	CCT intervention provided a significant but small increase in global cognitive function compared to that in the global cognitive function of the control groups. CCT intervention also resulted in a marginal improvement in domain-specific cognition compared to that in the control groups, with moderate heterogeneity. Subgroup analyses showed consistent improvement in global cognitive behavior in the CCT intervention groups.	- Overall - Episodic memory - EFs - Verbal memory - WM
Liang et al. [66]	CBT	No. of session: 4–54 Single intervention (min): — Total time: —	MCI Age: 66.9–78.3	13	CCT was significantly efficacious in NPI.	- Overall therapy effect
Martin et al. [63]	CBT	No. of session: — Single intervention (min): — Total time: —	HC; MCI Age: ≥ 60	36	Immediate and delayed verbal recall improved significantly through training compared to a no-treatment control condition, but the improvements observed did not exceed the improvement in the active control conditions	- Treatment versus active control: Delayed recall (MCI) - Treatment versus no contact: Delayed recall (MCI) - Treatment versus no contact: EFs(MCI) - Treatment versus active control: Immediate recall (MCI) - Treatment versus no contact: Visuospatial memory (HC) - Treatment versus no contact: Immediate recall (MCI) - Treatment versus no contact: Face-name delayed recall (HC) - Treatment versus no contact: Paired-associates (HC) - Treatment versus active control: Visuospatial (HC) - Treatment versus no contact: Face-name immediate recall (HC) - Treatment versus active control: Face-name delayed recall (HC) - Treatment versus active control: Paired-associates (HC) - Treatment versus active control: Delayed recall (HC) - Treatment versus active control: Face-name immediate recall (HC) - Treatment versus no contact: Short-term memory (HC) - Treatment versus active control: Short-term memory (HC) - Treatment versus no contact: Immediate recall (HC) - Treatment versus active control: Immediate recall (HC) - Treatment versus no contact: Delayed recall (HC)
Mewborn et al. [64]	CBT	No. of session: 1–180 Single intervention (min): ≥ 60 Total time: 1–270 h	HC; MCI Age: 63.8–85.1	97	Improvement of cognitive function	- Global cognitive functioning
Papaioannou et al. [38]	VR training	No. of session: 10–40 Single intervention (min): 25–100 Total time: 6–24 w	MCI Age: 62.8–85.3	16	Improvement of cognitive function	- Verbal function and language - EFs - Memory - General cognition
Shao et al. [36]	Computer-based intervention	No. of session: 10–75 Single intervention (min): 15–120 Total time: 4–24 w	HC Age: 68–82	12	Improvement of cognitive function, CCP valid complementary and alternative therapy for age-related cognitive	- EFs - Processing speed - Memory
Sherman et al. [20]	CBT	No. of session: 5–48 Single intervention (min): 30–120 Total time: 1–24 w	MCI Age: 63.1–76.3	32	Large effects for memory-focused training and moderate effects for multidomain interventions	- MT multidomain forms of intervention
Vaportzis et al. [17]	CBT	No. of session: — Single intervention (min): — Total time: 24–104 w	HC Age: ≥ 60	9	No effect of cognitive intervention	- EFs - WM - Processing speed
Wang et al. [67]	CBT	No. of session: — Single intervention (min): — Total time: 2–52 w	MCI Age: 67–86	11	Improvement of cognitive function	- WM - Immediate memory - Delayed memory - EFs - Global cognitive function
Wang et al. [68]	Cognitive training, CCT; cognitive stimulation; memory support system training; intensive cognitive rehabilitation program	No. of session: — Single intervention (min): — Total time: 4–52 w	MCI Age: 65.3–78.2	16	Improvement of cognitive function	- Global cognitive function
Xu et al. [69]	CBT	No. of session: — Single intervention (min): — Total time: 1–48 m	MCI Age: 61.7–85.8	14	Improvement of cognitive function	- Global cognitive function
Yan et al. [70]	VR-based intervention	No. of session: 12–36 Single intervention (min): 18–60 Total time: 6–36 w	MCI Age: 72–87.5	8	Improvement of cognitive function	- Memory - EFs - Attention - Global cognitive function
Zhang et al. [71]	CCT	No. of session: — Single intervention (min): — Total time: —	MCI Age: 65.9–78.5	18	Improvement of cognitive function	- WM - EFs - Memory - Global cognitive function
Zhong et al. [72]	VRCT	No. of session: 8–72 Single intervention (min): 18–100 Total time: 3–24 w	MCI Age: 62.8–87.2	17	Improvement of cognitive function	- Attention - MoCA - MMSE - Delayed memory - Immediate memory - EFs
Zhu et al. [73]	VRCT	No. of session: 10–40 Single intervention (min): 20–90 Total time: 3.5–60 h	MCI Age: 70.1–87.2	7	Improvement of cognitive function	- MoCA
Zuschnegg et al. [74]	Computer-based intervention	No. of session: — Single intervention (min): — Total time: 4–24 w	MCI Age: 66–76.6	17	Improvement of cognitive function	- MoCA

*Note:* CBT: cognitive-based intervention; h: hours; m: months; Min: minutes; w: weeks.

Abbreviations: CBCT, computer-based cognitive training; CCT, computerized cognitive training; EFs, executive functions; HC, healthy control; MCI, mild cognitive impairment; MMSE, mini mental state examination; MoCA, Montreal Cognitive Assessment; MT, memory training; SCD, subjective cognitive decline; VR, virtual reality; VRCT, virtual reality cognitive training; WM, working memory.

**Table 2:** Overview of the CCA score as a measure of primary study overlap.

Outcome	*k*	Primary studies	CCA^∗^
Global functioning	24	222	0.06
Memory	17	116	0.08
Executive functions	15	103	0.09
Attention	5	41	0.22
Visuospatial abilities	5	25	0.18
Processing speed	4	40	0.38
Language	4	9	0.61

*Note: k* = numbers of study.

^∗^Corrected covered area (CCA) is expressed in proportion.

## Data Availability

Data are available in the article's supporting information (Supporting Table 1b).

## Supporting Information

Supporting Information 1 Supporting Table 1a contains the ROBIS checklist items.

Supporting Information 2 The Supporting Table 1b contains the descriptive table of the articles included within the meta-analysis. Information included are author, year, intervention type and characteristics, intervention population, age, type of risk of bias adopted, outcome, ES, and results.
