# Supplementary material for: Evaluating the Effectiveness of Cognitive Interventions for Healthy and Mild Cognitive Impairment Adults: A Comprehensive Umbrella Meta-Analysis
Source: J Aging Res. 2025 Jun 16;2025:4397025. doi: 10.1155/jare/4397025 (PMC12185216; doi:10.1155/jare/4397025)
Supplement: Supporting Information 1 — Supporting Table 1a contains the ROBIS checklist items. [file 4397025.f1.docx]

**Supplement Table 1a.** ROBIS Checklist

| **ITEM_DOMAIN_1: study eligibility criteria** | |
| --- | --- |
| *Describe the study eligibility criteria, any restrictions on eligibility, and whether there was evidence that objectives and eligibility criteria were pre-specified* | |
| 1_1 | Did the review adhere to pre-defined objectives and eligibility criteria? |
| 1_2 | Were the eligibility criteria appropriate for the review question? |
| 1_3 | Were eligibility criteria unambiguous? |
| 1_4 | Were all restrictions in eligibility criteria based on study characteristics appropriate (e.g., Date, sample size, study quality, outcomes measured)? |
| 1_5 | Were any restrictions in eligibility criteria based on sources of information appropriate (e.g., publication status or format, language, availability of data) |
| **ITEM_DOMAIN_2: identification and selection of study** | |
| *Describe methods of study identification and selection (e.g., number of reviewers involved):* | |
| 2_1 | Did the review search an appropriate range of databases/electronic sources for published and unpublished reports? |
| 2_2 | Were methods additional to database searching used to identify relevant reports? |
| 2_3 | Were the terms and structure of the search strategy likely to retrieve as many eligible studies as possible? |
| 2_4 | Were restrictions based on date, publication format, or language appropriate? |
| 2_5 | Were efforts made to minimize errors in the selection of studies? |
| **ITEM_DOMAIN_3: data collection and study appraisal** | |
| *Describe methods of data collection, what data were extracted from studies or collected through other means, how risk of bias was assessed (e.g., number of reviewers involved), and the tool used to assess risk of bias:* | |
| 3_1 | Were efforts made to minimize errors in data collection? |
| 3_2 | Were sufficient study characteristics available for both review authors and readers to be able to interpret the results? |
| 3_3 | Were all relevant study results collected for use in the synthesis? |
| 3_4 | Was risk of bias (or methodological quality) formally assessed using an appropriate tool? |
| 3_5 | Were efforts made to minimize error in risk of bias assessment? |
| **ITEM_DOMAIN_4: Synthesis and findings** | |
| *Describe synthesis methods* | |
| 4_1 | Did the synthesis include all studies that it should? |
| 4_2 | Were all pre-defined analyses reported or their absence explained |
| 4_3 | Was the synthesis appropriate given the degree of similarity in the research questions, study designs and outcomes across included studies? |
| 4_4 | Was between-study variation (heterogeneity) minimal or addressed in the synthesis? |
| 4_5 | Were the findings robust, e.g. as demonstrated through funnel plot or sensitivity analyses? |
| 4_6 | Were biases in primary studies minimal or addressed in the synthesis? |
| **RISK OF BIAS IN THE REVIEW** | |
| *Describe whether conclusions were supported by the evidence* | |
| A | Did the interpretation of findings address all of the concerns identified in Domains 1 to 4? |
| B | Was the relevance of identified studies to the review's research question appropriately considered? |
| C | Did the reviewers avoid emphasizing results on the basis of their statistical significance? |
